# Iran's Approach to COVID-19: Evolving Treatment Protocols and Ongoing Clinical Trials

**DOI:** 10.3389/fpubh.2020.551889

**Published:** 2020-09-04

**Authors:** Ramin Rahmanzade, Reza Rahmanzadeh, Seyed MohammadReza Hashemian, Payam Tabarsi

**Affiliations:** ^1^Chronic Respiratory Diseases Research Center (CRDRC), National Research Institute of Tuberculosis and Lung Diseases (NRITLD), Shahid Beheshti University of Medical Sciences, Tehran, Iran; ^2^Faculty of Medicine, University of Basel, Basel, Switzerland; ^3^Neurologic Clinic and Policlinic, Departments of Medicine, Clinical Research, and Biomedical Engineering, University Hospital Basel and University of Basel, Basel, Switzerland

**Keywords:** Iran, COVID-19, SARS-CoV-2, antiviral therapy, infection prevention and control, public health

## Abstract

The coronavirus disease 2019 (COVID-19) pandemic is challenging the health care systems around the world and compelling them to timely share their strategies, tactics and experiences. Since mid-January, a huge volume of instructions has been released by Iran's Ministry of Health and Medical Education (MOHME) covering diverse aspects of disease control and prevention. In this study, we aimed to review the instructions published either before or after COVID-19's transmission to Iran to depict the clinical approach and therapeutics used in Iran to battle the current pandemic. We retrospectively gathered and critically reviewed all official situation reports, guidelines, guidance, flowcharts, protocols, recommendations and advice released by Iranian scientific, or administrative arms of action against COVID-19. The ongoing clinical trials approved by MOHME and registered to the Iranian Registry of Clinical Trials (IRCT) have been reviewed as well. Our study resulted in the following mainstays of Iran's approach to COVID-19: (i) active clinical screening; preferably on-line or on-phone, (ii) management of limited paraclinical resources; by using them as diagnostic tools rather than epidemiological, (iii) a trend toward outpatient care of mild-to-moderate cases; either confirmed or suspicious, with active scheduled follow-up, and (iv) avoidance of pharmacotherapy, as far as possible. The therapeutic and administrative instructions are still being actively updated with some recommendations different from the previous ones. Nevertheless, a common approach in the background could be detected, It seems that the instructions are conceptually in line with the first “National Guideline for 2019-nCoV” published on 20 January 2020. The screening has mainly been clinically oriented rather than being based on laboratory tests and MOHME seems to be following the approach of “early detection of symptomatic cases followed by early source control.”

## Introduction

Coronavirus Disease 2019 (COVID-19) first detected as unusual pneumonia in four Chinese patients on 26 December 2019, was quickly declared by the World Health Organization (WHO) as “Public Health Emergency of International Concern” on 30 January and, finally, as Pandemic on 11 March 2020 (1, 2).

The microbial cause of COVID-19 was identified on 7 January to be a coronavirus, at first called novel Coronavirus 2019 (2019-nCoV) and later SARS-CoV-2 by WHO (1, 2).

To timely being prepared against a possible epidemic of the novel coronavirus, Center for Disease Control and Prevention (CDC) of Iran's Ministry of Health and Medical Education (MOHME) released a comprehensive actionable guideline, named “National Guideline for 2019-nCoV” on 20 January 2020 and established thereby the primary framework for prevention, early detection and treatment of patients in the onward outbreak of the novel coronavirus based on WHO's Risk Communication and Community Engagement (RCCE) strategies, risk management strategies, infection prevention and control (IPC) strategies and internal instructions (3). A new, more detailed edition was released on 2 February, while still no case of COVID-19 was detected in Iran (4).

On 19 February 2020, while COVID-19 was reported in a total of 75,204 cases from 26 countries, MOHME officially announced the death of two old patients due to COVID-19 in Iran (5). Five days later, MOHME established the “Scientific Committee of COVID-19” aimed to release and continuously update an actionable “Diagnostic Therapeutic Flowchart for COVID-19,” abbreviated hereinafter as DTFC, as an appendix to the above-mentioned National Guidelines for 2019-nCoV. As of 6 June 2020, the flowchart that was first released on 25 February (DTFC1) has been updated six times (DTFC2-7) with several changes according to the national and international experiences (6–11).

Furthermore, specific guidance and protocols have been released for the clinical management of patients with COVID-19 in intensive care units (ICU) and for the pediatric and pregnant population (12–14).

In the past month, MOHME announced the appearance of second country-wide wave of COVID-19, which emerged in the regions spared by the first wave and spread to previously affected areas.

A summary of the main actions of WHO, MOHME, and “Iran's National Headquarter Against COVID-19” (INHAC) and a timeline of national and international events is outlined in Table 1.

**Table 1:** Timeline of main responses to the novel coronavirus pandemic at national and international level.

27. Dec 2019	1st report of 4 unusual pneumonia to local CDC in Wuhan, China
31. Dec 2019	1st report of pneumonia of unknown cause to the WHO China Country Office
04. Jan 2020	WHO publicly announced the pneumonia of unknown causes in Wuhan, China on social media
05. Jan 2020	WHO's 1st disease outbreak news advised against travel and trade restriction with China
07. Jan 2020	Novel coronavirus (nCoV-2019) identified
10. Jan 2020	- WHO's released “National capacities review tool for a novel coronavirus”: ongoing active monitoring and preparedness - WHO published an “Advice for international travel and trade”: no restriction for international traffic
12. Jan 2020	China publicly shared the genetic sequence of 2019-nCoV
13. Jan 2020	- The 1^st^ reported case of COVID-2019 outside of China (in Thailand) - WHO published an interim guidance for Risk communication and community engagement (RCCE), readiness and response to the novel coronavirus (2019-nCoV); updated on 26 January and 16 March 2020
17. Jan 2020	WHO released interim guidance for “laboratory testing for 2019-nCoV” (last update on 19 March)
20. Jan 2020	- MOHME released Iran's National Guideline for 2019-nCoV - WHO's field visit to Wuhan - WHO released an interim guidance for “home care of mild patients” (last update on 17 March 2020)
21. Jan 2020	China publicly released primers and probes used in rRT-PCR kits
22. Jan 2020	WHO's mission to China observed evidences of human-to-human transmission
24. Jan 2020	WHO published an update of “Advice for international travel and trade”: Advice for exit screening in countries with ongoing transmission and entry screening in countries without transmission
26. Jan 2020	Iran started screening at Point of Entry (PoE)
28. Jan 2020	- WHO released an interim guidance of Clinical management of severe acute respiratory infection (SARI) when 2019-nCoV is suspected−1^st^ report of limited human-to-human transmission outside China
30. Jan 2020	WHO declared the outbreak a Public Health Emergency of International Concern
01. Feb 2020	Iran's government officially banned flights from China
02. Feb 2020	MOHME released the 2^nd^ edition of Iran's National Guideline for 2019-nCoV
05. Feb 2020	Iran's government repatriated Iranian nationals from Wuhan, China (They have been isolated and closely monitored by MOHME)
11. Feb 2020	- WHO named the novel virus and the disease, SARS-CoV-2 and COVID-19, respectively - WHO convened a Research and Innovation forum on COVID-19 - WHO published key consideration for repatriation of travelers
19. Feb 2020	MOHME officially announced death of two patients due to COVID-19 in Iran
21. Feb 2020	MOHME release a guidance on environmental sanitation and safe burial
22. Feb 2020	- MOHME established the Scientific Committee of COVID-19 (SCC-19) - Iran's government closed schools and universities in the provinces affected by COVID-19
23. Feb 2020	Iran's government established Iran's National Headquarter against COVID-19
25. Feb 2020	SCC-19 released a “Diagnostic Therapeutic Flowcharts for COVID-19” (DTFC1)
26. Feb 2020	Interim guidance for IPC of COVID-19 in pregnant or breastfeeding mothers and in neonates and infants with mothers confirmed or suspicious to COVID-19
27. Feb 2020	Some provinces in Iran started to clinically screen the travelers at PoE
28. Feb 2020	Iran's National Headquarter against COVID-19 closed all schools around the country, decreased working hours and announced a nation-wide screening of travelers at PoE of all cities
02. Mar 2020	- WHO's field visit to Iran - SCC-19 released a DTFC for pediatric population - MOHME released an operative guidance for drug delivery and follow up in out-patient setting - MOHME officially advised against routine use of corticosteroid in COVID-19 - MOHME temporarily decreased the frequency of prenatal care visits
03. Mar 2020	- SSC-19 released DTFC2 and a protocol for management of critically ill patients with COVID-19 in intensive care units (ICU) - MOHME released interim guidance for follow up of COVID-19 patients treated in out-patient setting - MOHME released an action plan for care of COVID-19 patients in convalescent care facilities - MOHME released a guidance for in-patient care of pregnant women, confirmed or suspicious to COVID-19
04. Mar 2020	- MOHME launched a National public awareness campaign and released a detailed action plan
06. Mar 2020	- MOHME launched a National campaign against COVID-19 for active screening of COVID-19 and released a detailed action plan
07. Mar 2020	- MOHME released various therapeutic guidance for management of COVID-19 in patients with underlying chronic diseases e.g., cancer - MOHME released nutritional guidance for patients with COVID-19, treated in out-patient or in-patient settings - MOHME released guidance for telerehabilitation during viral epidemic
10. Mar 2020	- SCC-19 released DTFC3 - MOHME released a guidance for the use of Iranian traditional medicines in patients with COVID-19 - MOHME updated the operative guidance for drug delivery and follow up in out-patient setting, first released on 2 March 2020
11. Mar 2020	- WHO characterized COVID-19 as a pandemic
12. Mar 2020	- WHO's expert mission to Iran acknowledged Iran's strategies and comprehensive coordinated approach against COVID-19
13. Mar 2020	- WHO updated the interim guidance of Clinical management of SARI when 2019-nCoV is suspected, first released on 28. Jan 2020 - WHO launched COVID-19 Solidarity Response Fund
15. Mar 2020	- MOHME officially advised against routine use of oseltamivir in COVID-19 - MOHME released an additional protocol for management of COVID-19 in ICU to the previous guidance released on 03. Mar 2020
18. Mar 2020	- WHO launched the Solidarity Trial (2) - SCC-19 released DTFC4 - MOHME released an operative guidance for home care management of patients with mild COVID-19
25. Mar 2020	SCC-19 released DTFC5
30. Mar 2020	MOHME updated the action plan for care of COVID-19 patients in convalescent care facilities, first released on 3 March 2020
04. Apr 2020	MOHME released a guidance for clinical trials related to COVID-19
10. Apr 2020	MOHME launched the 2nd phase of National campaign against COVID-19: follow up of cases detected in 1st step and close-contact tracing of confirmed cases
14. Apr 2020	MOHME released an update of the protocol for management of critically ill patients with COVID-19 in intensive care units (ICU)
29. Apr 2020	SCC-19 released DTFC6
28. Jun 2020	SCC-19 released DTFC7

*In addition to the mentioned guidelines, flowcharts and protocols a significant number of advices, recommendations and guidance regarding environmental sanitation, social distancing, mental health and infection prevention and control (IPC) have been released by MOHME and other aligned, responsible authorities*.

Of note, the present narrative review is independent research trying to identify Iran's strategies against the pandemic of SARS-CoV-2 through scientific review of official documents released by responsible authorities.

## Iran's Internal Guidelines, Flowcharts, and Protocols on COVID-19: A Summary of Main Points

Since mid-January, different instructions and recommendations covering diverse aspects of the disease control and prevention have been released by INHAC, MHOME and their subdivisions in order to minimize the burden of the disease and the speed of its propagation.

The authors retrospectively gathered and critically reviewed all official situation reports, guidelines, guidance, flowcharts, protocols, recommendations and advice. The documents were collected through search of all actual or archived official documents released by MOHME or INHAC on the web since 26 December 2019.

Although the instructions are still being actively updated with some recommendations different from the previous ones, a common approach in the background could be detected. As noted on the cover page of DTFCs, these flowcharts are subjected to revisions based on new scientific findings or upcoming resource limitations.

The mainstays of the current approach consist of (i) Active clinical screening; preferably on-line or on-phone (ii) Management of limited paraclinical resources; by using them as diagnostic tools rather than epidemiological (iii) A trend toward outpatient care of mild-to-moderate cases; either confirmed or suspicious, with active scheduled follow-up (iv) Pharmacotherapy should be avoided as far as possible, rather in hospitalized patients or those who have been defined as high risk population.

### Active Clinical Screening

In January 2020, MOHME mainly tried to inform people about symptoms of COVID-19 and encourage patients with respiratory symptoms, especially those with a history of recent travel to China, to seek medical attention not too late. This approach of “early detection of symptomatic cases followed by early source control” was reinforced by the release of the National Guideline of 2019-nCoV on 20 January. Since late January, moreover, all passengers have been subjected to temperature screening upon arriving in Iran.

Despite these preparations, the transmission of the novel coronavirus was officially announced on 19 February and has been attributed to some passengers from China, who visited the crowded city Qom, which is one of the two main religious towns of the country. Of note, the visa-free policy of Iran for Chinese citizens may facilitate the travel and entry of Chinese to Iran at the beginning of pandemic in China.

Since 6 March 2020, the screening for COVID-19 has been entered a new phase. On this day, MOHME launched the “National Campaign Against COVID-19” and, thereby, established the three following bases for screening: (i) An electronic simple-to-deal portal, in which people fulfill a short web-based questionnaire with 6 questions. Finally, they receive a notification noting the possibility of having COVID-19 and some related recommendations. Moreover, in the case of being clinically suspicious to COVID-19 or being in contact with known cases of COVID-19, they will be automatically called by determined healthcare providers responsible for patients geographical sub-region, (ii) Telephone Screening through regional and sub-regional healthcare authorities via some publicly announced phone numbers, and (iii) Screening offices at selected and publicly announced medical centers and hospitals (15).

As of 5 August 2020, MOHME clinically screened more than 90% of Iran's population with above-mentioned methods as opposed to a total of 2,560,374 persons who were screened by the reverse transcriptase-polymerase chain reaction (RT-PCR) test.

As discussed above, the screening in Iran has mainly been clinically oriented and symptoms-based rather than being purely based on laboratory tests, an approach in agreement with the above-mentioned approach of “early detection of symptomatic patients followed by early source control.” However, getting equipped with enough laboratory resources e.g., diagnostic RT-PCR kits has activated the laboratory-based screening for a broader population of suspicious cases. On 10 April, MOHME launched the second phase of National Campaign Against COVID-19, mainly aimed to screen the people in close-contact with confirmed cases uning RT-PCR tests.

### Management of Limited Paraclinical Resources

The National Guidelines for 2019-nCoV mention that specimens, either from the upper or lower respiratory tract, should be taken from suspicious cases. Moreover, the RT-PCR should be repeated every 3–4 days in hospitalized patients until having two negative results with a time interval of at least 24 h. The first update of DTFC (DTFC2), released on 3 March 2020, mentioned that the RT-PCR for the detection of E gene (screening test) must be done for all hospitalized patients suspicious to COVID-19 and should be avoided in the outpatient setting. In addition, RT-PCR for the detection of N gene (confirmation test) should only be ordered in intubated patients with a positive RT-PCR for the E gene. The Next update (DTFC3), released on 10 March 2020, added that sampling and testing in the outpatient setting could be done in immunocompromised patients and health care providers suspicious to COVID-19.

Performing the RT-PCR test in clinically suspicious patients was first explicitly mentioned in DTFC6. It seems that the parallel increase in laboratory resources and reduction in the number of cases caused the extension of the eligible populations. In this line, all patients in close-contact with confirmed cases should also be tested based on DTFC6.

Blood tests of C-reactive protein (CRP) and complete blood count (CBC) have been noted that could be measured in afebrile patients without dyspnea presenting with respiratory symptoms, in the case of suspicion to COVID-19. However, DTFC6 substituted RT-PCR tests for these blood tests in clinically suspicious patients in the outpatient setting. DTFC7 restricted RT-PCR tests in outpatient setting only to patients or people with close contact (with a known COVID-19 case) who are older than 60 years-old, pregnant, or with a known medical history, which is a risk factor for COVID-19. Beside, based on DTFC7 all hospitalized cases need to perform RT-PCR test.

The role of imaging examinations as useful tools in the diagnosis of COVID-19 was not discussed until the DTFC1 was released. It could partly be resulted from the characteristic pulmonary involvement of COVID-19 compared to the SARS and MERS, which has been reported by other affected countries, especially China.

**Figure 1:**
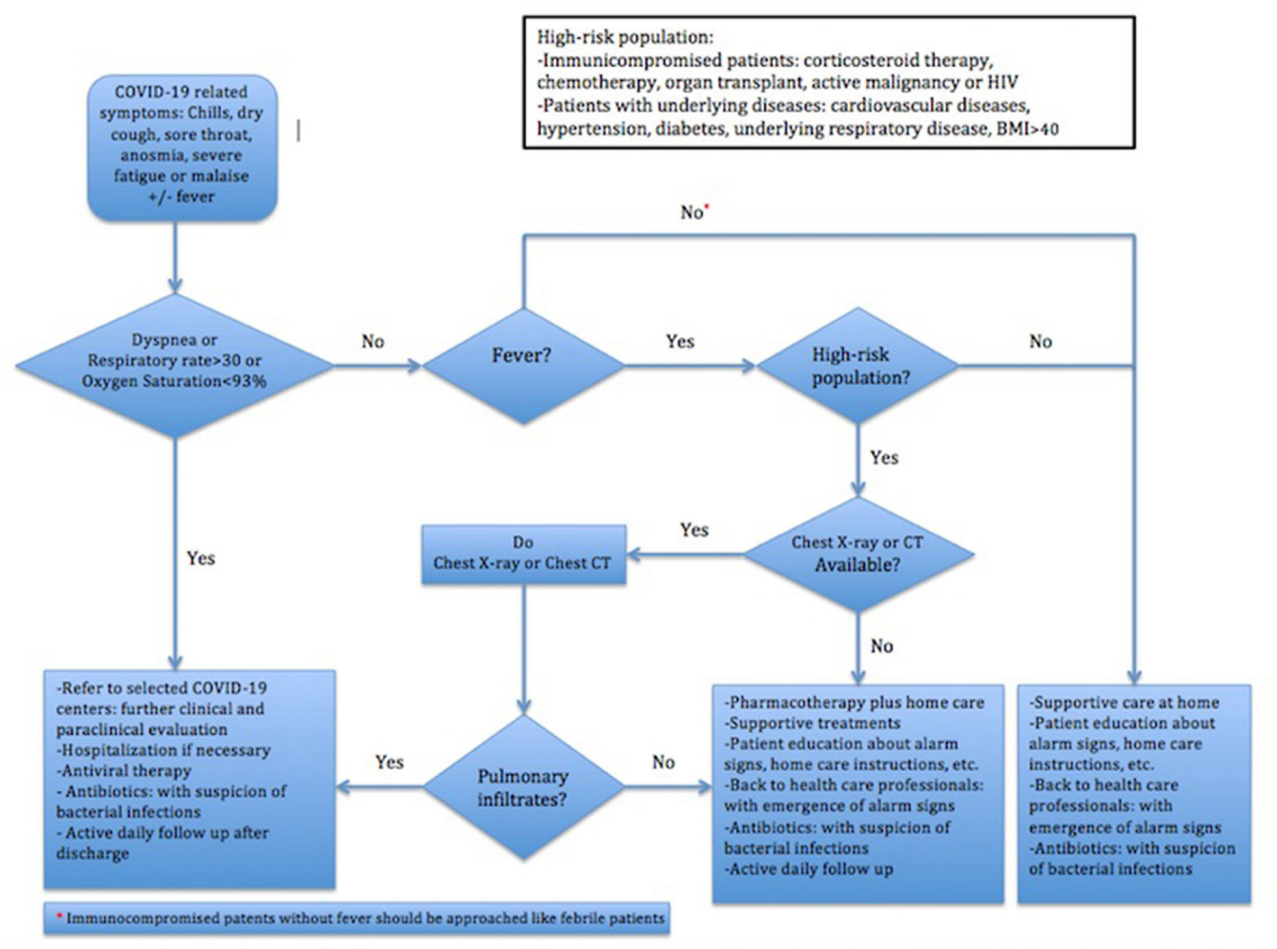
Iran's Flowchart of Clinical Approach to COVID-19 (10).

As shown in Figure 1, pulmonary imaging should be exploited in (a) febrile high-risk patients, defined in Figure 1, without dyspnea and (b) immunocompromised patients with clinical suspicion of COVID-19, independent of being febrile or not. The second group has been considered first in DTFC2. According to the DTFC2 and the later updates, COVID-19 compatible findings in chest CT scans might act as equivalent to the RT-PCR test in confirmation of the diagnosis of COVID-19 in clinically suspicious patients.

Although lung imaging has not been still considered as a tool in the follow-up of patients, a significant resolution of pulmonary involvement in imaging was included as an indispensable criterion for patient discharge from hospital in DTFC3-5, which has been removed in DTFC6.

### A Trend Toward Outpatient Care

The first guideline released on 20 January emphasized that patients with the mild disease could be cared at home and should be consulted to back to COVID-19 centers for early evaluation upon exacerbation of symptoms. In this line, DTFCs strongly recommended home care in all patients except the followings: (a) those presenting with respiratory symptoms having dyspnea or respiratory rate > = 30 per min or oxygen saturation <93%, (b) high-risk febrile patients with imaging findings compatible with COVID-19, and (c) immunocompromised patients suspicious to COVID-19. According to the “Operative guidance for home care management of patients with mild COVID-19,” patients should be consulted to enough rest, appropriate meals, high fluid intake and isolation. A detailed brochure, provided by MOHME, containing information about patient care at home, instructions of drug consumption, alarming symptoms and isolation will be given as well. In addition, all patients receiving outpatient care or therapy should be isolated for at least 14 days after the resolution of symptoms.

An active follow-up has been considered in all DTFCs as an indispensable part of the care of COVID-19 patients in outpatient settings. The follow-up is generally composed of (1) continuous self-monitoring and (2) scheduled surveillance by health care providers. According to the DTFC1, all patients treated in the outpatient setting should be daily followed up by health care providers in on-phone mode and upon emerging one of the following symptoms should be referred to hospitals: (a) dyspnea, (b) loss of consciousness, (c) continuation of fever, and (d) exacerbation of cough. However, the two latter criteria have been revised in DTFC2 to (c) a continuation of fever after 5 days from treatment start and (d) exacerbation of cough or productive cough, which are still valid.

The follow-up takes place through the Iranian Integrated Health portal, the so-called SIB®. The duration of daily telephone follow-up recommended by DTFC1 and DTFC2 has been explained in the next updates more detailed. According to the DTFC3-5, active telephone follow-up should daily be done in the first 5 days accomplished by a final follow-up 10 days after being registered as suspicious COVID-19 patient in SIB®. In DTFC6, the telephone follow-up should be done every 2 days until 14 days after registration in SIB®. In this regard, operative guidance was released on 2 March 2020, updated on 10 March, to precisely address the mechanism of drug delivery and follow-up in the outpatient setting. Based on these instructions, all patients receiving outpatient therapy will be registered in SIB® and have to be followed up as recommended in DTFCs.

Notably, convalescent care facilities have found a place in the care of patients with COVID-19 as an intermediate stage between inpatient and outpatient care settings and the role of these centers has been well-defined in an action plan released on 3 March 2020, updated later on 30 March 2020. Accordingly, patients with positive RT-PCR results, who: (a) are not hospitalized or (b) have been discharged earlier than 14 days after symptom onset or (c) might not receive enough care at home, should be referred to these centers.

### Therapeutics in Outpatient and Inpatient Settings: Regular Reconsideration

The national guideline released on 20 January 2020 and its single update on 2 February 2020, did not deal with therapeutics in detail. They generally recommended oxygen therapy, conservative rehydration and empirical antibiotic therapy in the setting of severe acute respiratory infection (SARI). Moreover, they are strongly against the routine use of corticosteroid in COVID-19 and recommend, also, the use of oseltamivir only when influenza is suspected. However, DTFCs have been mainly devised to be exploited as ready-to-use clinical action plans.

All versions of DTFC emphasized that COVID-19 could be managed in most patients without special antiviral or antibiotic therapies. In the outpatient setting, the recommendation of adjunctive medicines with possible beneficial effects against SARS-CoV-2 has been restricted to the following patient groups presenting with respiratory symptoms: (a) febrile high-risk patients without dyspnea with normal lung imaging (b) immunocompromised patients without dyspnea with normal lung imaging; independent of being febrile or afebrile.

In DTFC1-3, the treatment regimen in the outpatient setting consisted of oseltamivir 75 mg and hydroxychloroquine sulfate 200 mg (or chloroquine phosphate 250 mg) both twice daily for a minimum of five and a maximum of 14 days. In DTFC4-6 similar to the National Guidelines for 2019-nCoV, the indication of oseltamivir prescription has been restricted to those, in whom there is a virological or epidemiological clue of influenza infection.

In DTFC5-6, the proposed daily dose of hydroxychloroquine sulfate (or chloroquine phosphate) has been doubled on the first day of therapy and, also, the maximum duration of therapy has been reduced to 10 days. In DTFC7, the dosage for hydroxychloroquine sulfate (or chloroquine phosphate) administration remained the same as DTFC6, however was restricted to so-called “high risk population.”

In the inpatient setting, two different treatment regimens based on disease severity were proposed in DTFC1-4. According to the DTFC1-4, COVID-19 is considered to be very severe if at least one item of the followings is present: (a) loss of consciousness, (b) respiratory rate >= 24 per min, (c) systemic blood pressure <90/60 mm Hg, (d) multilobular infiltration on lung imaging, (e) persistent hypoxemia.

In patients not being classified as very severe, a combination of (i) hydroxychloroquine sulfate 200 mg (or chloroquine phosphate 250 mg) twice daily only on the 1st day, (ii) lopinavir/ritonavir 200 mg/50 mg two tablets twice daily for a minimum of five and a maximum of 14 days, which based on DTFC3-6 could be replaced by atazanavir/ritonavir 300/100 in the case of gastrointestinal intolerance or past history of cardiac arrhythmia. If atazanavir/ritonavir is prescribed, the hydroxychloroquine sulfate 200 mg twice daily should be continued for 5–14 days, (iii) oseltamivir 75 mg twice daily for 5–14 days was also recommended in DTFC1-3, not recommended in the later updates.

In patients, in whom the disease course was classified as very severe, ribavirin 1,200 mg daily based on DTFC1-2 and 2,400 mg daily based on DTFC3-4 plus the above-mentioned regimens was recommended. However, ribavirin has been removed from the proposed regimen in DTFC5-6. DTFC7 does not recommend hydroxychloroquine sulfate (or chloroquine phosphate) anymore for inpatient setting and recommends: (i) lopinavir/ritonavir 200/50 mg two tablets twice daily or atazanavir/ritonavir 300/100 once daily for a minimum of seven and a maximum of 14 days (ii) interferon-beta-1-a 250 microgram or interferon-beta-1-a 44 microgram subcutaneous every other day 5–7 days.

Indeed, the DTFC5-7 do not deal anymore with critically ill patients and referred the clinical management of this group of patients to a separate “protocol for the management of critically ill patients with COVID-19 in intensive care units (ICU) (12).”

Therefore, DTFC5-7 recommends only hydroxychloroquine sulfate 200 mg (or chloroquine phosphate 250 mg) two tablets twice daily on the 1st day and then one tablet twice daily for 7–14 days in the inpatient setting. In addition, concomitant therapy with lopinavir/ritonavir or atazanavir/ritonavir (two tablets twice daily for 7–14 days) is a dispensable part of standard regimen in DTFC5-7 and might be ordered at the discretion of the responsible clinicians.

According to the ICU protocol, lopinavir/ritonavir or atazanavir/ritonavir should be prescribed in ICU-admitted patients with a respiratory rate >= 24 per min or SpO_2_ = <80–85% (12). This protocol recommended ventilatory support suggested by WHO (16), the Surviving Sepsis Campaign (17), and Marini and Gattinoni (18). It considers oxygen therapy if SpO_2_ <90% and recommends intubation in COVID-19 patients as early as one item of the followings is present: (a) persistent hypoxemia (PaO_2_ <60 mmHg or SpO_2_ <85%) following 1–2 h application of non-invasive ventilation or 30–60 min usage of high-flow devices (b) moderate to severe respiratory acidosis (PaCO_2_ >= 60 mmHg or PH = <7.25) (c) Respiratory rate >= 36 per minute (d) Hemodynamic instability (mean arterial pressure (MAP) <60 mmHg without response to the fluid therapy), and (e) loss of consciousness.

## Registered Clinical Trials

As of 4 August 2020, a total of 305 clinical trials have been registered to the Iranian Registry of Clinical Trials (IRCT) (19). As a part of WHO's SOLIDARITY trial, 16 centers in Iran are involved in a large five-arm randomized controlled trial with a target sample size of 3,000, The recruitment phase has been completed in May 2020. SOLIDARITY trial (IRCT20200405046953N1) aims to evaluate the safety and efficacy of four different medicines including Remdesivir, chloroquine/hydroxychloroquine, lopinavir/ritonavir, and interferon plus lopinavir/ritonavir on COVID-19. The enrolled patients will receive these medications in conjunction with the local standard regimens.

Among registered trials, 17 randomized trials evaluate or compare the safety and efficacy of different antivirals including sofosbuvir, sofosbuvir/ledipasvir, sofosbuvir plus velpatasvir, sofosbuvir plus daclatasvir, ribavirin, lopinavir/ritonavir, favipiravir, umifenovir, and remdesivir in the treatment of COVID-19 (IRCT20151227025726N14, IRCT20200322046833N1, IRCT20200128046294N2, IRCT20200324046850N2, IRCT20200318046812N1, IRCT20100228003449N29, IRCT20171122037571N2, IRCT20130812014333N145, IRCT20200328046882N1, IRCT20200421047155N1, IRCT20130812014333N145, IRCT20080901001165N46, IRCT20200403046926N1, IRCT20200328046886N1, IRCT20200406046968N3, IRCT20180725040596N2, IRCT20200428047228N1). The largest randomized trial on antivirals (IRCT20200318046812N1) is currently recruiting patients from 11 centers with a target sample size of 324. It is designed to compare the therapeutic efficacy of hydroxychloroquine plus favipiravir with a combination of hydroxychloroquine plus lopinavir/ritonavir in COVID-19.

A single-arm non-controlled trial (IRCT20171122037571N2) with a target sample size of 120 evaluates the safety and efficacy of remdesivir in COVID-19 patients. The control group is treated with the standard regimen and the intervention group concomitantly receives remdesivir and the standard regimen for 5 days. The recruitment phase has been completed in May 2020.

As the humoral immunity is vastly involved in antiviral immunity, some groups suggested the potential beneficial role of blood products in the treatment of COVID-19. Eighteen trials have been designed to evaluate the efficacy and safety of convalescent plasma or intravenous immunoglobulin in COVID-19 (IRCT20200325046860N1, IRCT20200310046736N1, IRCT20151228025732N53, IRCT20181104041551N1, IRCT20200325046859N1, IRCT20200317046797N3, IRCT20200409047007N1, IRCT20200413047056N1, IRCT20200416047099N1, IRCT20200406046968N2, IRCT20200501047258N1, IRCT20200418047116N1, IRCT20150808023559N21, IRCT20200414047072N1, IRCT20120215009014N353, IRCT20200404046948N1, IRCT20150808023559N20, IRCT20200328046882N1). The largest of them (IRCT20200325046860N1), is currently recruiting patients with severe COVID-19 from four COVID-19 centers with a target sample size of 200 and an expected recruitment completion on 20 August 2020. A trial with a target sample size of 45 (IRCT20200310046736N1) aims to compare the safety and efficacy of convalescent plasma with the plasma-derived immunoglobulin-enriched solution in COVID-19 patients.

Seven trials have been registered to assess the efficacy of interferon (INF) ß-1a or 1b (IRCT20080901001165N53, IRCT20200406046968N3, IRCT20160118026097N3, IRCT20190804044429N1, IRCT20150914024017N1, IRCT20100228003449N28, IRCT20100228003449N27, IRCT20151227025726N12). The recruitment phase of the four latters has been completed with a targeted sample size of 40, 30, 30 and 20, respectively. A trial (IRCT20190804044429N1) with a sample size of 70 compares the efficacy of the standard regimen with the combination of the standard regimen and INF ß-1b. The recruitment phase completed in May 2020. The largest one (IRCT20080901001165N53) with a sample size of 100 is studying the efficacy of INF ß-1a nasal spray.

Among 10 registered trials in the recruiting phase, which evaluate the prophylactic or therapeutic efficacy of chloroquine/hydroxychloroquine in COVID-19 (IRCT20130917014693N10, IRCT20100228003449N30, IRCT20120826010664N6, IRCT20200718048129N1, IRCT20100228003449N3, IRCT20130306012728N8, IRCT20080901001165N51, IRCT20190122042450N4, IRCT20151222025660N2, IRCT20200405046958N1), the first one is the largest with a target sample size of 100 patients and an expected date of recruitment completion of 8 June 2020. The latter (IRCT20080901001165N51), a randomized trial, studies the efficacy of hydroxychloroquine nasal spray in 80 patients.

Nine trials are aimed to study the safety and efficacy of mesenchymal stem cell therapy in COVID-19, all with a small sample size of 5–10 (IRCT20200418047121N2, IRCT20200413047063N1, IRCT20130306012728N8, IRCT20190717044241N2, IRCT20200217046526N2, IRCT20200217046526N1, IRCT20140911019125N6, IRCT20200325046860N2, IRCT20140528017891N8). The recruitment phase of the two latters with a sample size of 5 and 10 has been completed.

Five trials (IRCT20151227025726N13, IRCT20150303021315N17, IRCT20200501047258N1, IRCT20200406046968N3, IRCT20130306012728N8) evaluate the safety and efficacy of Tocilizumab in COVID-19. The latter, a multicentric study with a target sample size of 500 will expectedly complete the recruitment phase in July 2020.

The use of corticosteroids in COVID-19 is still controversial. WHO's interim guidance released on 13 March 2020 and Iran's National guidelines for 2019-nCoV are explicitly against routine use of corticosteroids (3, 4, 16).

To evaluate the efficacy of corticosteroids, six trials have been registered (IRCT20200324046852N1, IRCT20200404046947N1, IRCT20200204046369N1, IRCT20200406046963N1, IRCT20170210032478N3, IRCT20120215009014N354). These are randomized trials investigating efficacy of corticosteroid administration, either oral or inhalational or intravenous, as adjunctive therapy to the standard regimens.

The recruitment phase of the two first trials with a sample size of 30 and 40 patients, respectively, has been completed.

A large multicentric trial (IRCT20200318046812N2), with a target sample size of 906, recruits as of 10 April patients from four centers for 1 year. This study aims to evaluate the safety and efficacy of three therapeutic interventions with the following regimens: “Hydroxychloroquine + Azithromycin + Naproxen + Prednisolone” or “Hydroxychloroquine + Azithromycin + Naproxen” regimens or “Hydroxychloroquine + Kaletra.”

ACE2, a negative regulator of the renin-angiotensin-aldosterone system (RAAS) works as the SARS-CoV-2 entry receptor. In this line, medications with activity on RAAS e.g., ACE inhibitors and angiotensin receptor blockers (ARB) could be of therapeutic interest.

A randomized controlled trial (IRCT20180802040678N4) with a target sample size of 100 patients evaluates the efficacy of losartan 25 mg twice daily on the treatment of COVID-19. The recruitment phase has been completed in May 2020. Similar to SARS-CoV, the spike protein priming of the SARS-CoV-2 during entry to the alveolar epithelial cells occurs through the serine protease TMPRSS2 (20). A randomized clinical trial (IRCT20200317046797N1) comparing a treatment group receiving camostat mesylate, a serine protease inhibitor, plus the standard regimen with a control group receiving only the standard regimen completed its recruiting phase in May 2020.

A total of 98 clinical trials has been registered to the IRCT to evaluate the efficacy of diverse herbal or traditional Iranian medicines, supplements or vitamins e.g., high dose Vit C (IRCT20190917044805N2) and Vit D3 (IRCT20200324046850N1).

As of 5 August 2020, a total of 314,786 confirmed, 17,617 death and 272,535 recovered COVID-19 cases have been reported by Iran.

## Conclusion

The presented narrative review tries to identify the mainstays of Iran's approach to COVID-19. As almost all of the national instructions released by responsible authorities are in Persian, this review may contribute to the global data sharing in the era of COVID-19. The presented review found that the instructions are conceptually in line with the first “National Guideline for 2019-nCoV” published on 20 January 2020. The screening has mainly been clinically oriented rather than being purely based on laboratory tests and MOHME seems to be following the approach of “early detection of symptomatic cases followed by early source control.”

## Author Contributions

RaR devised the project and the main conceptual idea. RaR and ReR gathered, reviewed the documents, took the lead in writing the manuscript, and wrote it in consultation with SH and PT. SH and PT revised the first draft of manuscript. RaR drew the diagram. ReR, SH, and PT made the table. All authors proofed the final draft of manuscript.

## Conflict of Interest

The authors declare that the research was conducted in the absence of any commercial or financial relationships that could be construed as a potential conflict of interest.

## Abbreviations

WHO: world health organization
2019-nCoV: novel coronavirus 2019
SARS-CoV-2: severe acute respiratory syndrome coronavirus 2
COVID-19: Coronavirus Disease 2019
RCCE: Risk Communication and Community Engagement
IPC: infection prevention and control
SCC-19: Scientific Committee of COVID-19
DTFC: Diagnostic Therapeutic Flowchart for COVID-19
INHAC: Iran's National Headquarter Against COVID-19
MOHME: Iran's Ministry of Health and Medical Education
RT-PCR: reverse transcriptase- polymerase chain reaction
ICU: intensive care unit
INF: interferon
SpO2: saturation of peripheral oxygen
PaO2: partial pressure of oxygen.
